# Development of marker-free transgenic pigeon pea (*Cajanus cajan*) expressing a pod borer insecticidal protein

**DOI:** 10.1038/s41598-021-90050-8

**Published:** 2021-05-18

**Authors:** Snehasish Sarkar, Souri Roy, Sudip K. Ghosh

**Affiliations:** 1 Advanced Laboratory for Plant Genetic Engineering (ALPGE), IIT-Kharagpur, Kharagpur, India; 2 Department of Biotechnology, IIT Kharagpur, Kharagpur, 721302 India

**Keywords:** Plant sciences, Plant biotechnology, Molecular engineering in plants

## Abstract

Pigeon pea, a grain legume of the semiarid tropics, is a rich source of high-quality protein. The productivity of this pulse is seriously affected by lepidopteron insect pests. To generate a sustainable insect-resistant plant, synthetically prepared bioactive key constituents of a crystal protein (Syn Cry1Ab) of *Bacillus thuringiensis* were expressed in pigeon pea under the guidance of a tissue-specific promoter of the RuBP carboxylase/oxygenase small subunit (*rbcS*) gene. Regenerated transgenic plants with the *cry1Ab* expression cassette (*cry1Ab-lox-bar-lox*) showed the optimum insect motility rate (90%) in an in vitro insect bioassay with second instar larvae, signifying the insecticidal potency of Syn Cry1Ab. In parallel, another plant line was also generated with a chimaeric vector harbouring a *cre recombinase* gene under the control of the CaMV 2 × 35S promoter. Crossing between T_1_ plants with a single insertion of *cry1Ab-lox-bar-lox* T-DNA and T_1_ plants with moderate expression of a *cre* gene with a linked hygromycin resistance (*hptII*) gene was performed to exclude the bialaphos resistance (*bar*) marker gene. Excision of the *bar* gene was achieved in T_1_F_1_ hybrids, with up to 35.71% recombination frequency. Insect-resistant pigeon pea plants devoid of selectable marker genes (*syn* Cry1Ab- *bar* and *cre-hptII*) were established in a consecutive generation (T_1_F_2_) through genetic segregation.

## Introduction

Pigeon pea is one of the most popular protein-rich grain legumes consumed as a pulse in the semiarid tropics (SAT) region^1^. Compared to other pigeon pea-producing countries, India contributes 90% to global production. This crop has social, economic and medicinal importance in developing countries^2^. It can grow in a variety of environmental conditions throughout the year due to its adaptive nature. This adaptive quality minimizes the cultivation cost, which ultimately increases the profit of marginal farmers^3^. The seed of this plant contains approximately 20–22% protein, mostly sulfur-containing amino acids, viz., cysteine and methionine, which is almost three times more than that of cereals^4^. Moreover, seeds also contain carbohydrates (57.3–58.7%), crude fibres (1.2–8.1%), and lipids (0.6–3.8%)^5^. In the Caribbean region, pigeon pea is consumed as a green vegetable due to its balanced nutritional quality^6,7^. Despite its high demand, the productivity of pigeon pea has increased only 1% during the past several years. This caused a serious scarcity in the per capita availability of this pulse, mainly in India^8^. The major factors for this low production are biotic and abiotic stresses and a lack of cultivation management practices. One of the most serious biotic stresses associated with pigeon pea cultivation is the infestation of *Helicoverpa armigera*, a lepidopteran pest^9,10^. Its larvae attack green parts such as the leaves, flowers, and pods of growing plants, resulting in considerable damage of approximately 40–50% and annual yield losses equivalent to 400 million US$ worldwide^11^. Farmers frequently use different kinds of chemical pesticides to fight this pest. Although this method has immense potential to control pests even in adverse conditions, it contaminates food and food products with insecticide residues^12^ and allows for the emergence of secondary pest problems. In addition, the unaccountable use of these chemicals is also responsible for the development of insecticide-resistant pests, which makes it more complex to address this problem only through chemical control. Elite pigeon pea cultivars with insect resistance could be produced through conventional breeding methods to overcome this pest problem. Unfortunately, this method has not been successful due to the incompatibility with wild species and the presence of limited genetic variation in the cultivated germplasm^6^. Recently, the plant genetic engineering approach has shown immense potential to overcome this type of difficulty. Genes conferring resistance to insect pests have been introduced successfully in a wide array of crop plants^13^. Hence, the development of insect-resistant cultivars through transgenic approaches has become an effective alternative to the integrated pest management program^14,15^. Efficient regeneration and transformation systems, a prerequisite to introducing any novel trait for successful development of transgenic plants, have been achieved in pigeon pea^4,16,17,18,19^. Some success in the development of pod borer-resistant pigeon pea/chickpea has already been demonstrated by using synthetic *cry1E-C*, c*ry1Ab*, *cry1Ac*, and chimaeric *cry1AcF* as insecticidal genes^11,20,21^; however, there is inadequate evidence validating transgenic pigeon pea events in terms of the stability of protein expression and the rate of insect mortality^22^. Despite these preliminary successes, more positive events with adequate expression of the toxin gene resulting in a more significant impact on pod borer-resistant plants under field conditions are needed. The marker gene, which is required for screening transformed plants under in vitro conditions, raises public and regulatory concerns^23^; therefore, removal of the marker gene should also be taken into account to prevent gene flow and introgression. One of the straightforward ways to eliminate the possibilities of marker gene transfer is the excision of the marker gene after the development and selection of transgenic plants using a site-specific recombinase system (e.g., *cre-lox* system). This could be possible in a hybrid plant resulting from a cross between two lines. Establishment of an efficient crossing procedure is essential to create successful hybrid plants though crossing; however, it is tedious and requires specialised skills along with the knowledge of various stages of flower development ^24,25,26^.

Considering all the previous reports and requirements, an effort has been made to develop a marker gene-free transgenic pigeon pea plant expressing the *cry1Ab* gene for resistance against the legume pod borer *H. armigera*.

## Results

### Development of a green tissue-specific, insect resistance *syn cry1Ab* and *cre* line in pigeon pea

#### Generation of the *cry1Ab* line

To achieve sustainable resistance against the lepidopteran insect pest *H. armigera*, the synthetically prepared bioactive core element of the *cry1Ab* gene of *B. thuringiensis* was expressed in pigeon pea. The toxin protein was expressed in a tissue-specific manner and showed insecticidal potency against *H. armigera* under laboratory conditions. The results are elaborated in the following section.

#### Generation of pLBRCAb transgenic pigeon pea lines harbouring the *syn cry1Ab* transgene

The cotyledonary node of the Asha variety was cocultivated with *A. tumefaciens* EHA105 harbouring the pLBRCAb vector, with three experimental attempts. *Agrobacterium*-mediated transformation and regeneration were performed following the established protocol reported by Sarkar et al^17^. Transformed regenerated plants were selected using medium containing bialaphos (4 mg/L) as a selection agent. Under selection pressure, ~ 16% of the regenerated plants showed resistance to bialaphos and further developed into healthy plants after 50 days of recovery. Surviving plants were rooted under selection pressure.

#### Transformation efficiency of T_0_ transformants

Drug-resistant T_0_ plants were further analysed by dot blot to determine the transformation efficiency of the transformed genes. For this, three sets of experiments were performed, wherein a total of 27 plants (T_0_) showed resistance against the selection medium out of 300 infected explants. Among these 27 plants, 10 showed positive results for the dot blot analysis, indicating 3.33% transformation efficiency (Table 1).

**Table 1:** Transformation efficiency of pigeon pea (T_0_): Three independent sets of *Agrobacterium*-mediated transformation and their transformation efficiency.

Set	No of explants infected by *Agrobacterium*	Bialaphos resistant shoots (T_0_)	Dot blot positive	Trans. efficiency (%)
Control	75	0	0	0
1	100	9	3	3
2	100	11	5	5
3	100	7	2	2
	Total = 300	Total = 27	Total = 10	Mean = 3.33

#### Progeny analysis of transgenic pigeon pea lines harbouring *syn cry1Ab*

**Figure 1:**
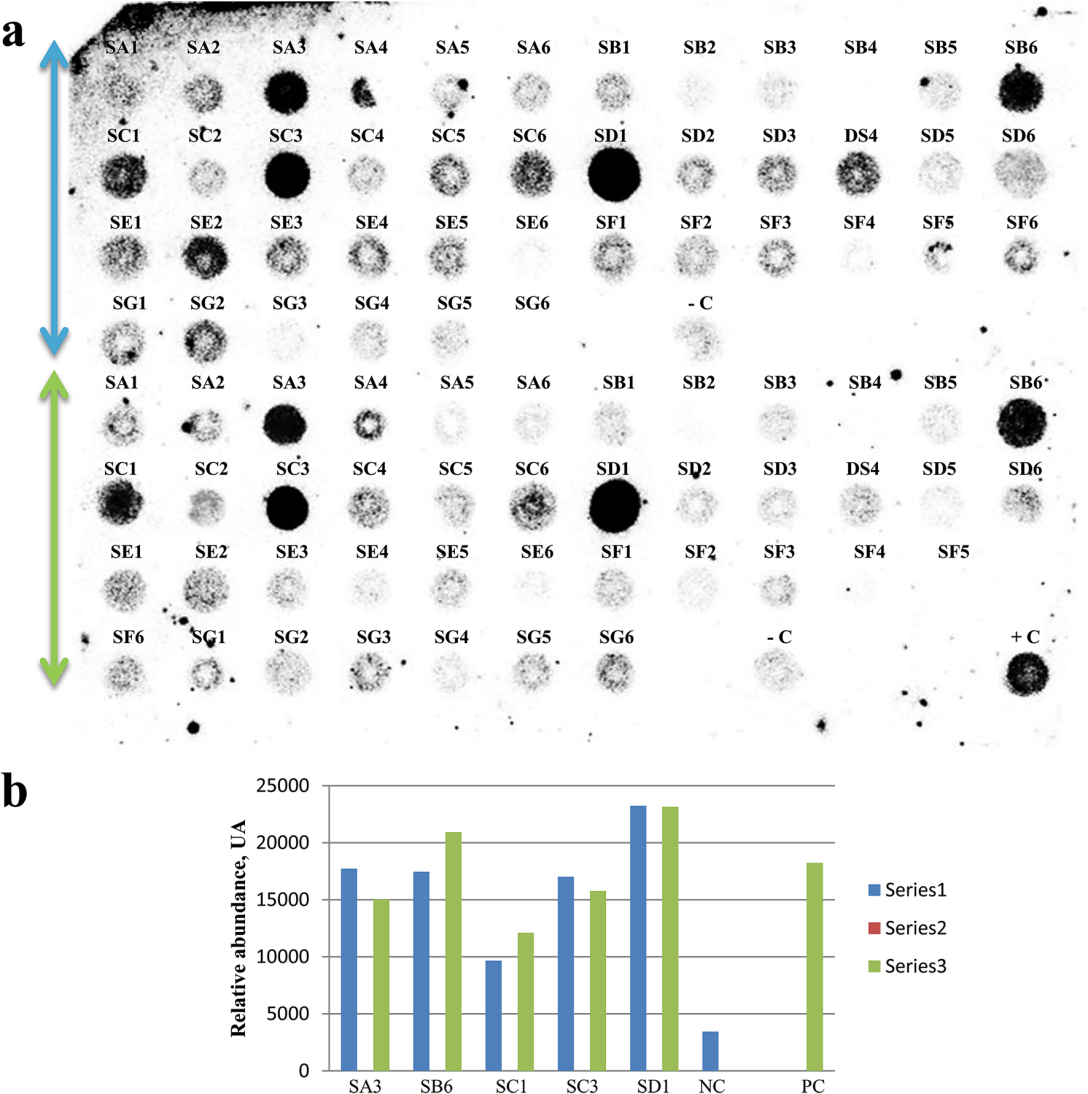
(**a**) Dot blot hybridization of the *cry1Ab* gene in the T_1_ generation: Six randomly selected T_1_ plants from 7 individual events (T_0_ plants) were analysed. A replica blot of the genomic DNA of 42 individual plants was prepared and hybridized with the 800 bp PCR amplified *cry1Ab* gene as the probe. In this replica blot, SA3, SB6, SC1, SC3, and SD1 showed a positive result, where –C is the untransformed control and + C is the positive control. (**b**) Graphical representation of the densitometry analysis of dot blots with two replicas by ImageJ Software (Version: 1.50, https://imagej.nih.gov/ij/download.html).

**Figure 2:**
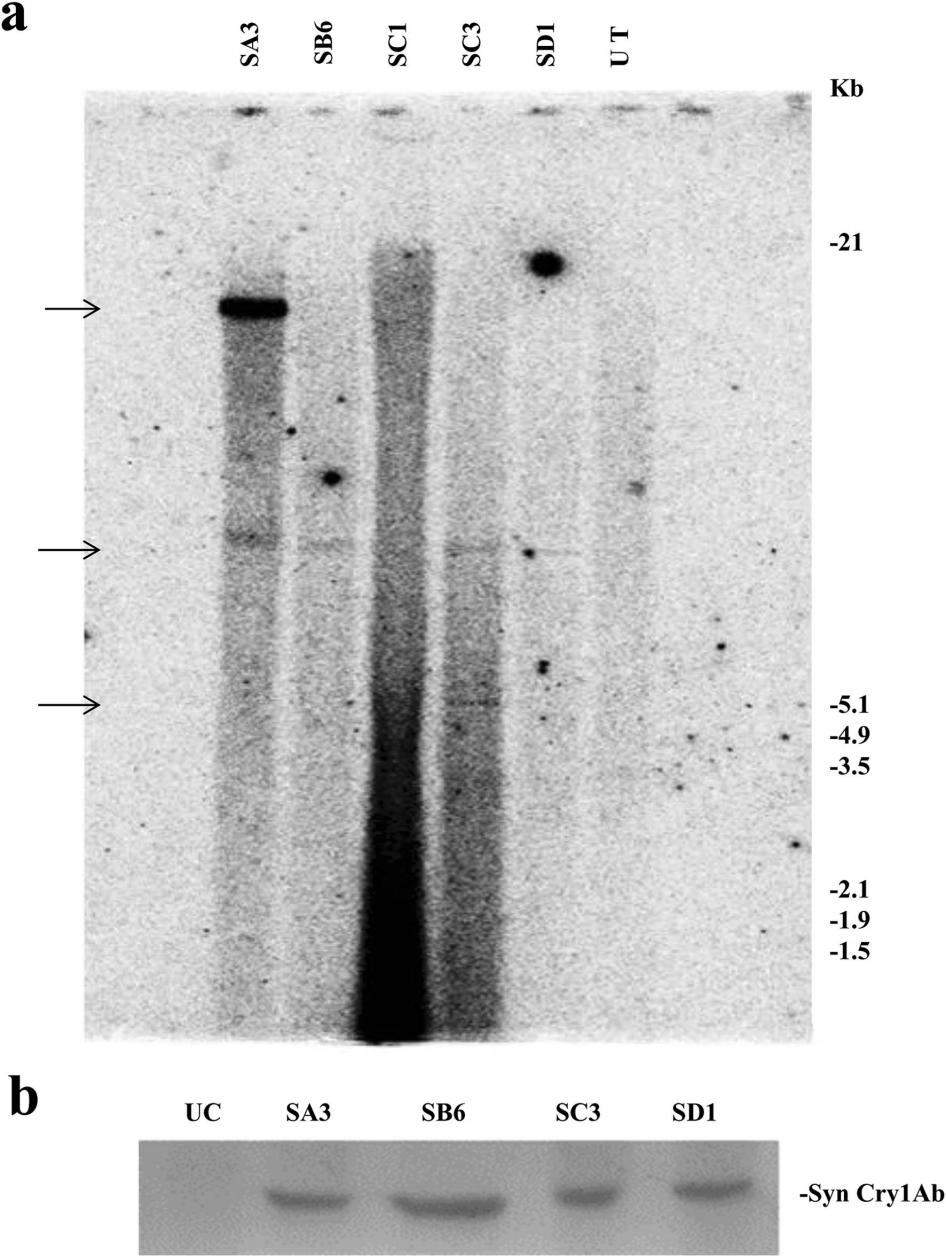
(**a**) Southern blot hybridization of the *cry1Ab* gene in the T_1_ generation: Genomic DNA of dot blot-positive T_1_ plants was digested with *Hind*III. The blot was hybridized with the 800 bp PCR-amplified *cry1Ab* gene (α-^32^[P]dCTP labelled)) as the probe. Lanes labelled SB6 and SD1 showed a single integration, SA3 and SC3 show a double integration, and UT shows the untransformed control. Approximate molecular weight markers are indicated. (**b**) Western blot analysis of the Syn Cry1Ab toxin protein in T_1_ progeny plants with the anti-Cry1Ab antibody: Lane UC: Untransformed control. Lanes SA3, SB6, SC3, and SD1: Transgenic lines.

Out of several putative transformants generated using pLBRCAb, only 7 healthy plants were selected for further analysis after stringent screening. Six individual progenies of each of the 7 T_0_ plant lines (total 42 plants) were randomly selected to carry out dot blot hybridization. Among these plants, 5 (SA3, SB6, SC1, SC3, and SD1) showed positive results (Fig. 1a). Densitometry analysis of dot blots was also carried out by ImageJ software (Fig. 1b) for comparison with the positive and negative controls. SD1 and SC1 plants showed the highest and lowest intensity, respectively. Furthermore, the presence of the *cry1Ab* gene in the T_1_ generation was confirmed by Southern blot analysis (Fig. 2a). Of these 5 individual transformants selected through dot blot analysis, 4 plants contained *cry1Ab* based on Southern blot analysis, among which 2 showed a single integration and 2 showed a double integration of the *cry1Ab* gene, while the untransformed control plant did not show any band. This experiment provided evidence of stable integration of the transgene and its transfer to consecutive generations.

#### Expression analysis of the ***syn cry1Ab*** gene in T_1_ transgenic lines

All four Southern-positive transgenic pigeon pea lines of *syn cry1Ab* (SA3, SB6, SC3, and SD1) were subjected to expression analysis. Expression of the *syn cry1Ab* gene in four transgenic lines was analysed using an anti-Cry1Ab antibody by western blot (Fig. 2b); distinct bands were observed in all plants. No Cry1Ab protein band was observed in the untransformed control line.

#### Entomocidal activity of Syn Cry1Ab toxin present in the T_1_ transgenic pigeon pea

**Figure 3:**
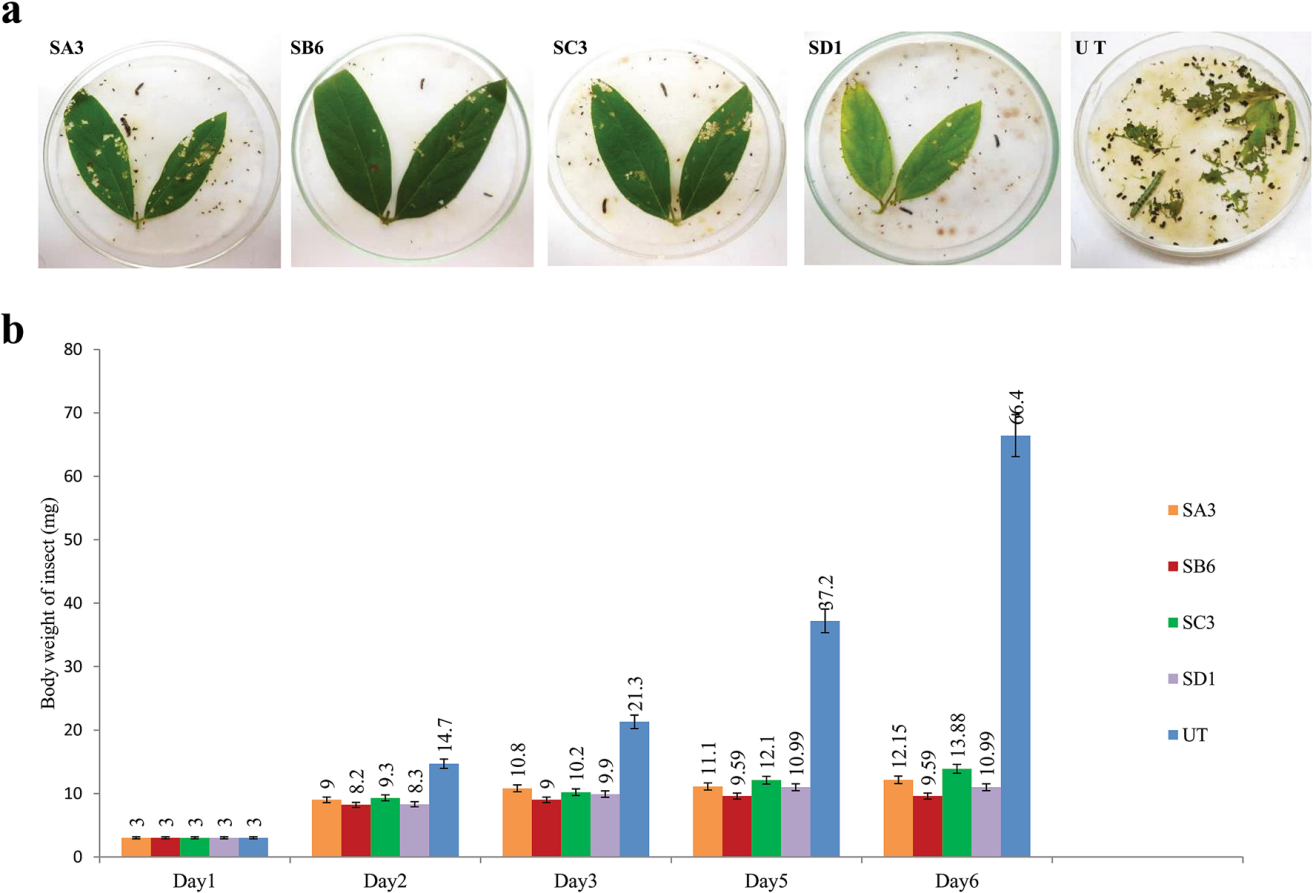
In vitro insect (*H. armigera*) feeding assay: The T_1_ transgenic plants with 5 replica experiments were carried out with two insects for each individual plant. (**a**) SA3, SB6, SC3, and SD1 are transgenic plants that showed a positive result, and UT is an untransformed negative control plant. (**b**) Graphical presentation of insect weight loss during the feeding assay in comparison with the untransformed control.

Transgenic plants were tested for insecticidal activity. Fully opened young, fresh leaves of four T_1_ transgenic pigeon pea lines were tested through an in vitro insect feeding bioassay to confirm the insecticidal activity of Syn Cry1Ab toxin against second instar larvae of *H. armigera*. The young leaves of the untransformed plants were used as a negative control. The transgenic plants showed very little damage after feeding by insects, while massive damage was observed with the untransformed control line (Fig. 3a). Extensive feeding of leaf tissue (> 90%) by the larvae was observed for untransformed control plants, and larvae were healthy, active, and showed regular developmental cycles. Larvae fed on transgenic plants SA3, SB6, SC3, and SD1 showed a 70–90% mortality rate with a 79.1–85.5% bodyweight reduction (Table 2). The gradual decreases in larval body weight after 6 days of incubation in the insect feeding bioassay are graphically presented in Fig. 3b.

**Table 2:** Mortality and bodyweight reduction of the larvae fed leaf tissues of T_1_ transgenic lines after 6 days of incubation.

Plant No	Total no of insect	No of insect die after 6 days	Mortality (%)^#^	Body weight reduction (%)^#^
SA3	10	7	70	81.7
SB6	10	9	90	85.5
SC3	10	8	80	79.1
SD1	10	9	90	83.4
Control	10	0	0	0

^#^Mortality and body weight was calculated on 10 insects; 5 replica experiments were carried out by two insects for an individual plant.

#### Segregation analyses of the ***bar*** gene in T_1_ progeny

The segregation pattern of the *bar* transgene through seeds in the T_1_ progeny was monitored by germination on the selection medium. Randomly selected seeds of each of the four T_0_ transformants were screened for germination in 4 mg/l bialaphos-containing medium alongside seeds of untransformed plants. It was observed that some of the T_1_ seeds did not grow after germination and gradually turned brown in the presence of bialaphos. Most of the T_1_ seedlings could grow in the presence of bialaphos. The ratio of bialaphos resistant:sensitive seedlings was scored after 4 weeks, and it was observed that the ratio was close to the expected ratio (3:1) in each case, indicating a Mendelian mode of inheritance for the monohybrid cross (Table 3). The plants that survived on the screening medium confirmed the stable inheritance of the *bar* transgene, whereas the untransformed control plants ceased to germinate and gradually turned brown even after 10 days on the same medium. The germinated plantlets were transferred to pots in the greenhouse for growth and further analysis. Since the *bar* gene is linked to the *cry1Ab* gene, the inheritance pattern of the *cry1Ab* gene is expected to be the same as that of the *bar* gene.

**Table 3:** Segregations analysis of *bar* gene in the T_1_ progeny of transgenic pigeon pea plant lines based on bialaphos resistance (R) and susceptibility (S).

Plant line	Number of T_1_ seeds tested	Bialaphos^R^	Bialaphos^S^	Observed ratio	χ^2^ value	*p* value
SA3	18	13	5	2.6:1	0.074	0.7855
SB6	23	17	6	2.8:1	0.014	0.9042
SC3	20	15	5	3:1	0.00	1.00
SD1	22	16	6	2.6:1	0.061	0.8055

The expected ratio was 3:1, *p* < 0.05, χ^2^ = 3.841.

#### Generation of *cre* lines

To eliminate the selectable marker (*bar*) gene from *syn cry1Ab* transgenic pigeon pea lines through Cre/*lox*-mediated recombination, *cre* pigeon pea lines were generated. To catalyse recombination, crossings were performed between the *cre* plant line and *syn cry1Ab* plant line, which carries *lox-bar-lox*. Finally, superfluous *cre* and *hpt*II genes were excluded through consecutive genetic segregation. The results are elaborated in the following sections.

#### Generation of transgenic pigeon pea lines harbouring the *cre recombinase* gene

**Figure 4:**
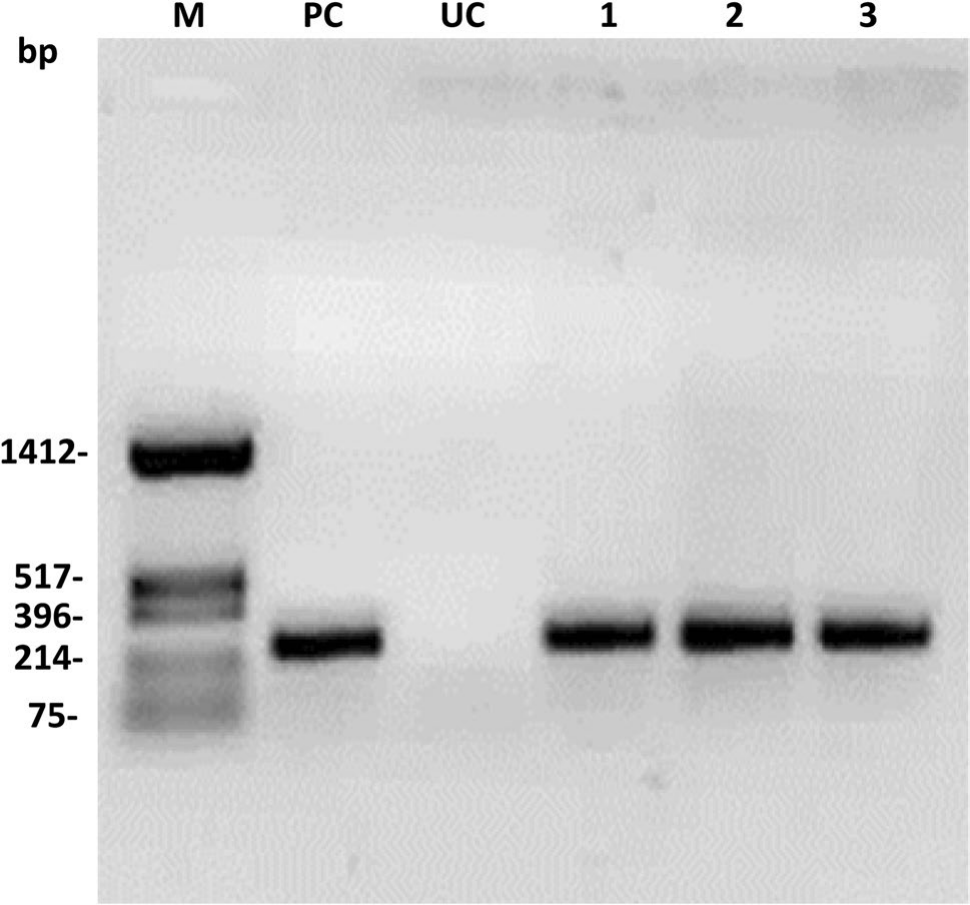
PCR analysis of putative T_0_ transformants with *cre* gene-specific primers: UT: Untransformed control, PC: Positive control and M: *Hin*fI digested pUC18 as a molecular weight marker.

The pigeon pea transformants with pHC (H: *hpt*II and C: *cre*) were generated following the *Agrobacterium*-mediated pigeon pea transformation protocol in Asha varieties. A total of 270 explants were cocultivated with *Agrobacterium* EHA105 containing pHC. Only 27 regenerated plants were retrieved after stringent hygromycin selection (50 mg/L) (Table 4). Among them, 13 plantlets showed a *cre* gene-specific PCR amplification product (275 bp) with *cre* gene-specific primers (CRFP2/CRRP2) (Supplementary Table S1). Representative PCR amplification of the *cre* gene is shown in Fig. 4.

**Table 4:** Generation of transgenic pigeon pea lines harboring pHC through *Agrobacterium*-mediated transformation.

Experiments	No. of explant infected	No. of Hygromycin^R^ plants	PCR positive plantlets	Transformation efficiency (%)
1st	90	7	4	4.44
2nd	90	6	3	3.33
3rd	90	14	6	6.66
Total	270	27	13	4.81

#### Seed transmission of the ***cre*** gene through T_1_ generation and progeny analysis

A containment condition was created to prevent cross-pollination and allow T_0_ transgenic pigeon pea plants to self-pollinate, and T_1_ seeds were harvested. T_1_ seeds from representative transgenic plants were initially screened by growing on antibiotic (50 mg/L hygromycin)-supplemented media. The sensitive seedlings ceased to grow and gradually died, whereas resistant seedlings showed healthy growth. The data regarding transgene (*hpt*II) segregation in T_1_ progenies were evaluated by the χ^2^ test. The results showed that the *hpt*II gene segregated closely in a Mendelian fashion with a 3:1 segregation ratio (Table 5).

**Table 5:** Segregations analysis of T_1_ progeny of pHC containing transgenic pigeon pea based on hygromycin resistance (R) and susceptibility (S).

Plant line	No of T_1_ seeds tested	Seedlings		Observed ratio	Chi-square (χ^2^) value	*p* value
		Hyg^R^ (Green)	Hyg^S^ (Etiolated)			
CA	25	17	8	2.12:1	0.6533	0.41893
CB	38	28	10	2.8:1	0.0351	0.85138
CC	32	23	9	2.5:1	0.1660	0.68315

The expected ratio was 3:1, *p* < 0.05, χ^2^ = 3.841.

#### Southern blot analysis for the confirmation of the presence of the ***cre*** gene in the T_1_ line

**Figure 5:**
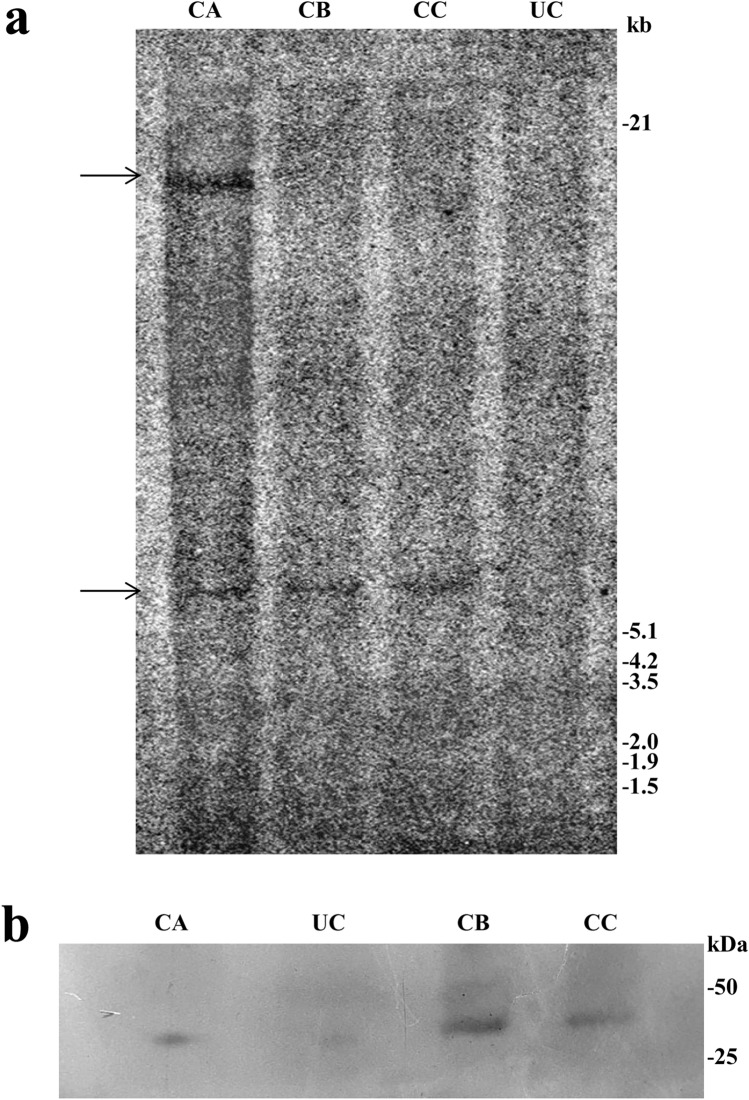
(**a**) Southern blot analysis of PCR-positive plants in T_1_ using the *cre* gene as the probe. Genomic DNA was digested with *Hin*dIII. Lane UC: Genomic DNA from the untransformed control line, and lanes CA, CB and CC: genomic DNA of transformed plants. Approximate molecular weight markers are indicated. (**b**) Western blot analysis of T_1_ transgenic lines with the polyclonal Cre recombinase antibody raised in rabbit for the detection of *cre recombinase* gene expression. Lane UC: Untransformed control. Lanes CA, CB, and CC: Transgenic plant lines.

The integration of the *cre recombinase* gene into the pigeon pea transformants was further confirmed by Southern blot analysis. Randomly chosen PCR-positive seeds of transformants grown in the presence of hygromycin selection were selected for Southern blot analysis. The genomic DNA of T_1_ plants was digested with *Hind*III restriction endonuclease, and hybridization was performed with an α-[32P] dCTP radiolabelled probe of the *cre* gene (1202 bp). The developed blot of three transformed lines (CA, CB, and CC) showed transgene integration (Fig. 5a). Among these three plants, the presence of a single insertion of the *cre* gene was observed in two plants, viz., CB and CC, while double integrations were observed in one plant, viz., CA. No band was detected for the untransformed control (UC) plant.

#### Western blot analysis of the T_1_ line to determine the presence of Cre recombinase

The expression of the *cre* gene was analysed by western blot analysis. Total leaf protein was isolated from these three Southern blot-positive plants (30 days old). The total soluble protein of transgenic plants and untransformed controlled plants was loaded and separated on a 12% SDS-PAGE gel. Upon western blot analysis using the anti-Cre antibody (Product no. ab41104, Abcam, UK), distinct bands were observed in all transgenic pigeon pea plants (Fig. 5b). The three individual plants showed specific expression of the 38 kDa Cre protein. No band was detected in the case of the untransformed control (UC) plant.

### Elimination of selectable marker genes from *syn cry1Ab* pigeon pea lines through Cre/*lox*-mediated recombination and consecutive genetic segregation

#### Crosses between T_1_***syn cry1Ab*** and ***cre*** lines

To initiate the crossing between T_1_ *syn cry1Ab* and *cre* lines for the production of marker-free insect-resistant pigeon pea, we first identified the appropriate time of crossing. The outcomes of the findings are discussed in the following sections.

#### Identification of the appropriate timing for crossing in pigeon pea

Flowers at different stages were collected to observe the morphological development of the stigma and anther in the process of flower development. A flower fully covered with calyx was designated stage zero, whereas blooming of the flower was divided into four stages up to fully bloomed, depending upon the morphological appearance (Supplementary Fig. S3). The anther and stigma of the respective stages of flower development were separated and observed under a microscope. The stagewise development of anthers and stigmas and their functions are represented in Supplementary Fig. S4. In the present observation, it was found that stigma at stage 3 (Supplementary Fig. S4, 3a) had the highest pollen receptivity. In stage 2 (Supplementary Fig. S4, 2b), it was observed that the anthers started to release pollen, and no pollen was attached to the stigma (Supplementary S4, 2a) until that time. Stage 2 was found to be an appropriate stage for the emasculation of the anther, as it begins to open for discharging pollen. Stage 3 (1–3 PM) is also suitable for pollination in the emasculated flower; in this stage, stigmas show the highest pollen receptivity and become sexually active for fertilization. After that, the receptivity of the stigma decreases continuously over time.

#### The crossing of parental pigeon pea lines and screening of hybrid plants

Two independent T_1_ progeny plants (SB6 and SD1) harbouring pLBRCAb [considered the female parent (**♀)**] were crossed with a T_1_ progeny plant (CC) harbouring pHC [considered the male parent **(♂)]** with moderate *cre* gene expression. The hybrid progenies obtained from the cross between T_1_ pHC and the pLBRCAb line were named T_1_F_1_. The consecutive generation of the hybrid line was designated T_1_F_2._ All the seeds of two independent crossing experiments were harvested, air-dried, and stored. The hybrid seeds were surface sterilized and germinated on MS medium in the presence of 50 mg/L hygromycin. Drug-resistant seedlings were transferred to the greenhouse and allowed to grow to maturity for further detection of selectable marker gene (*bar*) elimination.

#### PCR-based analysis of marker gene elimination following Cre/*lox*-mediated recombination

**Figure 6:**
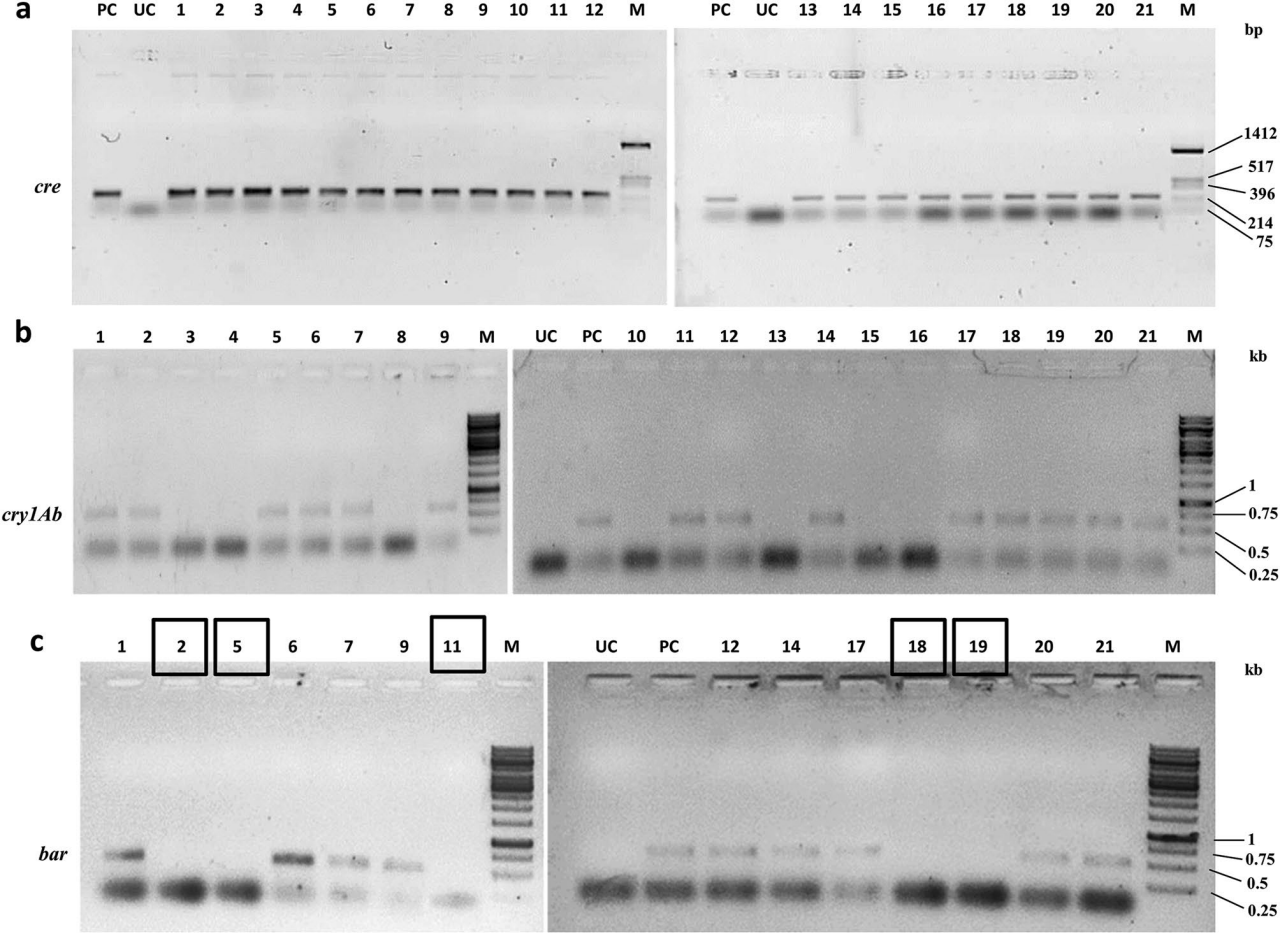
PCR analysis of the 21 T_1_F_1_ hybrid line for Cre-mediated *bar* gene elimination. (**a**) PCR amplification of hygromycin-selected progenies of line SB6CC with *cre-*specific primers. Lane PC: PCR from the pHC plasmid as a positive control; lanes 1–21: 21 T_1_F_1_ hybrid progenies. The 275 bp amplicon represented the *cre* recombinase. (**b**) PCR with *syn cry1Ab*-specific primers of 21 *cre-*positive plants of the T_1_F_1_ hybrid line SB6CC. Fourteen plants showed amplification of a representative 720 bp region of the *syn cry1Ab* gene. Lane PC: pLBRCAb as a template. (**c**) PCR with *bar*-specific primers of 14 *syn cry1Ab-* and *cre-*positive plants of the SB6CC line. Lanes 1–14: T_1_F_1_ hybrid plants, where plants no. 2, 5, 11, 18 and 19 showed the absence of *bar*. Lane PC: PCR from pLBRCAb; the 552 bp amplicon represents the *bar* gene. Plant lines boxed in black show successful elimination of the *bar* gene. Lane M in (**a**), *Hin*fI-digested pUC18 DNA as a molecular weight marker. Lane M in (**b**) and (**c**), GeneRuler 1 kb DNA Ladder, Thermo Scientific. Lane UC represents PCR results from the genomic DNA of an untransformed plant. All figures of each independent gel are separated by a white border.

The T_1_F_1_ seeds (SB6CC) were collected separately from two independently crossed events (Table 6) and germinated on selection medium containing hygromycin (50 mg/L) to eliminate possible azygous plants for *hpt*II and the linked *cre* transgene. Forty-two hybrid seeds from the SB6CC line were randomly selected for initial hygromycin screening. Among the hybrid seeds, 21 seeds grew into plantlets under selection pressure (Table 6). These initially screened hybrid plants were further screened (verified) by separate PCRs with *cre*, *syn cry1Ab*, and *bar* gene-specific primer pairs. The representative data from hybrid line SB6CC are shown in Fig. 6a–c. Among these 21 progeny plants, 14 plants were found to be positive for both *cre* and *syn cry1Ab* genes; 7 plants were positive for only the *cre* gene (Fig. 6a,b). As expected, no plant harbouring only the *syn cry1Ab* cassette was obtained, as these plants did not contain the *hpt*II gene and thus were excluded from the screening process. Marker gene elimination among the 14 *syn cry1Ab-* and *cre*-positive hybrid plants of SB6CC was further verified through PCR using *bar* gene-specific primers (Fig. 6c). This PCR result showed five *bar*-negative plants, as the *bar* gene was expected to be eliminated through recombination due to the presence of the *cre recombinase* gene. Based on this finding, the frequency of Cre/*lox*P-mediated recombination was calculated to be 35.71% in the case of hybrid line SB6CC. Similarly, the other hybrid line SD1CC showed a 33.33% recombination frequency, with an average recombination frequency for these two lines of 34.52%.

**Table 6:** Crosses between T_1_ *syn cry1Ab* and *cre* lines, and estimation of the frequency of Cre/*lox* mediated marker elimination.

Crossing events	T_1_ parent (♀)	T_1_ parent (♂)	Hybrid line ID	Total lines	*Cre* positive lines	*Syn cry1Ab* and *cre* positive lines	*bar* negative lines	Recombination frequency (%)*
1	SB6	CC	SB6CC	42	21	14	5	35.71
2	SD1	CC	SD1CC	38	23	12	4	33.33
Total				80	44	26	9	34.52

*Recombination frequency = (No. of *bar* negative plants/ No. of *syn cry1Ab* and *cre* positive plants) × 100%.

#### Establishment of completely selectable marker-free T_1_F_2_ transgenic pigeon pea plants

**Figure 7:**
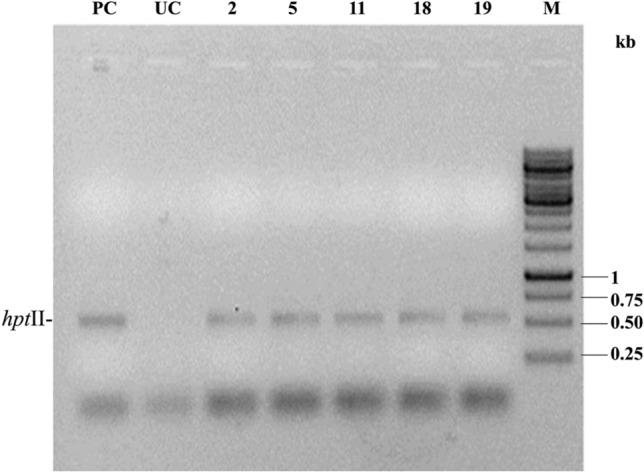
PCR analysis of the five T_1_F_1_ *bar-*negative, *cre-*positive plants with *hpt*II gene-specific primers. PC: Amplification of pHC as a positive control; UC: Untransformed control. M: GeneRuler 1 kb DNA Ladder, Thermo Scientific.

**Figure 8:**
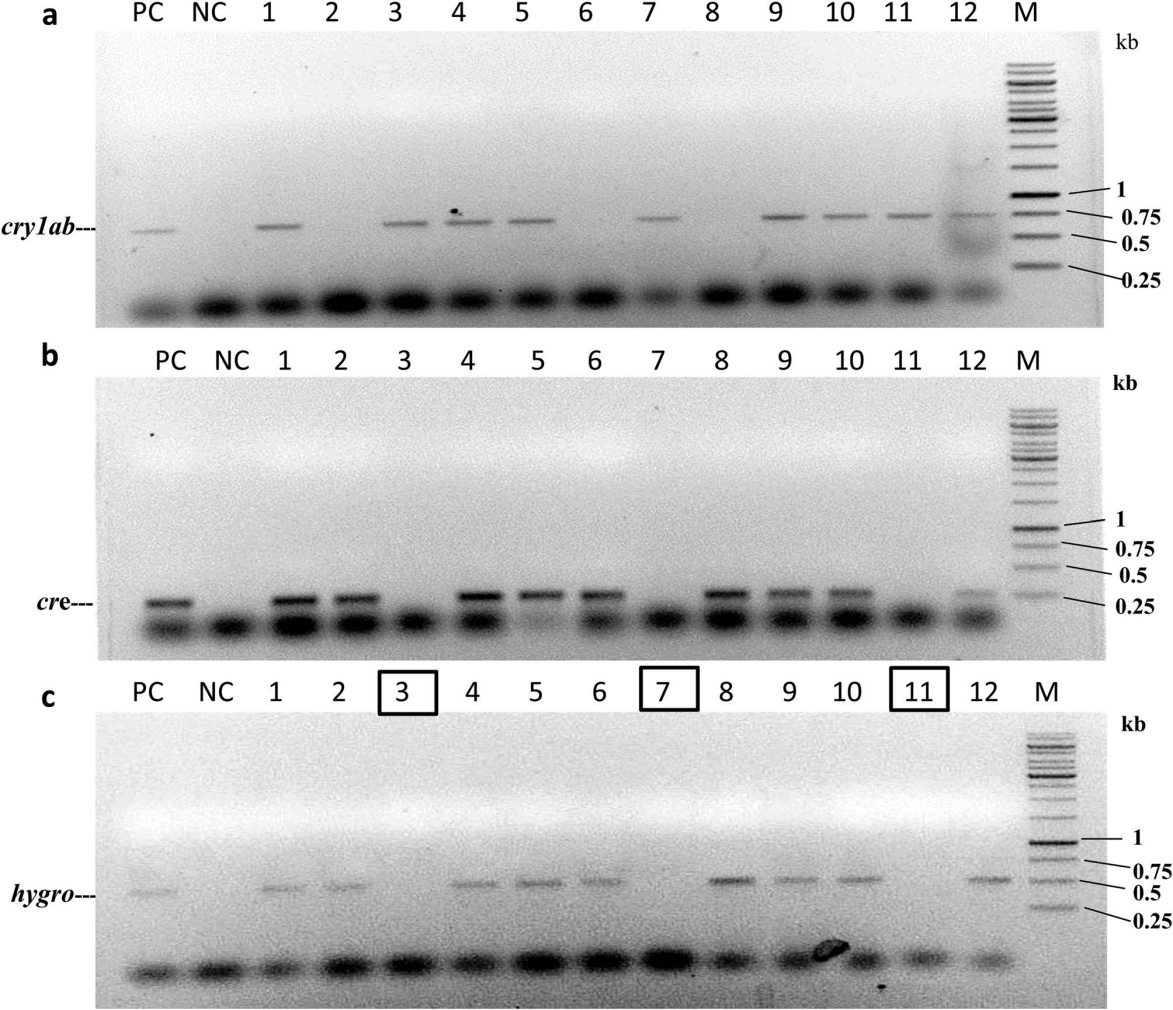
PCR analysis of 12 randomly selected *bar*-negative SB6CC T_1_F_2_ plants to establish complete elimination of the *cre-hpt*II gene. (**a**) PCR with *syn cry1Ab*-specific primers, (**b**) PCR with *cre*-specific primers, and (**c**) PCR with *hpt*II-specific primers. PC: positive control (amplification from *syn cry1Ab* or *cre* gene containing expression constructs); UC: no amplification from the untransformed control line. MF (Marker Free): represents T_1_F_2_ plants with complete elimination of all the selection markers (*bar*) and redundant transgenes (*cre-hpt*II linked gene) with retention of the toxin gene. M *Hin*fI-digested pUC18 as a molecular weight marker.

As the *bar*-negative T_1_F_1_ plants are *cre* positive, there is a high possibility that they still bear *hpt*II as a linked gene. PCR-based screening with *hpt*II gene-specific primers supported this fact (Fig. 7). Consequently, some of the T_1_F_1_ plants underwent self-fertilization to obtain T_1_F_2_ progeny plants and were analysed for complete excision of selectable marker genes through genetic segregation. Twelve T_1_F_2_ progenies of the *bar*-negative line SB6CC (19) were analysed for the presence of *syn cry1Ab*, *cre*, and *hpt*II genes. Six plants [T_1_F_2_ plant SB6CC (19)- 1, 4, 5, 9, 10,12] contained *syn cry1Ab*, *cre* and *hpt*II genes, three plants [SB6CC (19)- 2, 6, 8] showed the presence of *cre* and *hpt*II, and three plants [SB6CC- 3, 7, 11] were found to contain only *syn cry1Ab* without the presence of the *cre* or *hpt*II gene (Fig. 8a,b,c). Thus, the elimination of the selectable marker gene (*hpt*II) and linked transgene (*cre*) in T_1_F_1_ plants was performed by genetic segregation in the consecutive generation (T_1_F_2_). Therefore, these three identified pigeon pea plants are now considered completely marker-free transgenic *syn cry1Ab* lines.

**Figure 9:**
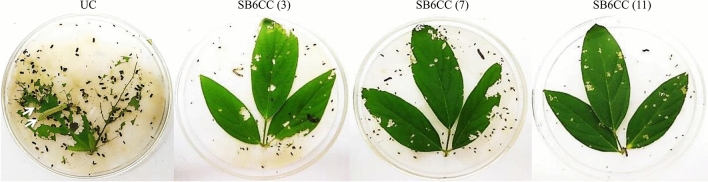
In vitro insect (*H. armigera*) feeding assay of T_1_F_2_ marker-free transgenic plants of the SB6CC line: Transgenic plants with 3 replica experiments were carried out by second instar larvae for each individual plant. Insect feeding assay of SB6CC(3), SB6CC(7) and SB6CC(11) transgenic plants with two insects showed a positive result; UC is an untransformed control plant (leaf), and the white arrow shows live and healthy larvae.

Finally, the insecticidal activity of Syn Cry1Ab toxin in T_1_F_2_ marker-free transgenic plants of the SB6CC line was analysed by an in vitro insect feeding assay. In this experiment, young transgenic leaves were incubated with second instar larvae and compared with insects fed untransformed control leaves. The transgenic plants showed very little damage, while massive damage was observed for the untransformed control line (Fig. 9). Extensive feeding of leaf tissue (> 90%) by the larvae was observed for the untransformed control plants, and all the larvae were healthy, active, and showed regular developmental cycles. Larvae fed on transgenic plants SB6CC(3), SB6CC(7), and SB6CC(11) showed > 90% mortality rates with drastic body weight reduction. The data were collected after 6 days of incubation in an insect feeding bioassay.

## Discussion

Agricultural production loss due to insect pest damage is a severe problem worldwide. Extensive time and effort has already been spent to address this issue. The massive use of chemical pesticides to counter these losses not only poses a significant threat to the environment but also requires significant costs. This expense often cannot be afforded by marginal farmers in developing countries. Accordingly, that focus has shifted towards advances that have already been made in the field of genetic engineering regarding the generation of insect-resistant crops. Even though GM or Bt crops are controversial, biotechnologically produced plants have gained importance over those produced via conventional crop improvement through breeding, mainly for the development of insect resistant crops^27^. Thus, the comparatively less time-consuming transgenic technology would be the best choice for plant improvement over conventional breeding to address the growing problem of food scarcity. The development of transgenic plants expressing the insecticidal protein gene of *B. thuringiensis* has been one of the powerful techniques to develop insect-resistant crop plants offering protection against insect damage. To date, more than 30 insect-resistant crop plants have been developed using the *Bt* toxin gene.

However, in reality, an efficient regeneration and transformation method for the development of stable transgenic plants plays an important role. Pigeon pea is a leguminous plant that is not readily amenable to tissue culture for in vitro plant regeneration. However, by adopting a protocol for multiple shoot development from embryonic meristematic tissue of pigeon pea followed by lateral branching reported by Sarkar et al^17^, this constraint was suitably resolved.

The present experiment was performed according to our previously reported regeneration and transformation protocol^17^. The *Agrobacterium*-mediated transformation of the pLBRCAb cassette resulted in the generation of 27 prospective bialaphos-resistant plants (T_0_). Ten transformants were shown to be positive in terms of transgene integration with 3.33% transformation efficiency, as revealed by dot blot hybridization (Table 1). Furthermore, progeny analysis was performed on 7 well-grown/surviving transgenic plants (T_0_). Six drug-selected plantlets (T_1_) of seven T_0_ plants were randomly selected and subjected to dot blot analysis (Fig. 1a), which confirmed the integration of candidate genes. Single representative positive plants of each event (T_1_) were subjected to further Southern blot analysis, which revealed the integration pattern of the *cry1Ab* gene.

Integration of the *cry1Ab* transgene into the genome of pigeon pea was found to vary in number and loci (Fig. 2), indicating that all were separate events except SB6 and SD1, which might be the same event or siblings. In our present study, Southern-positive plants in the T_1_ generation were considered stably integrated transgenic events; western blot experiments revealed that these transgenic plants produced transgenic protein. The survival of T_1_ seeds in bialaphos-containing media indicated that the transgenes segregated according to the Mendelian 3:1 ratio and were transmitted to the next generation (Table 3). This observation also revealed that a single copy of the transgene could be integrated at a single site or two copies of the transgene could be integrated very close together on a chromosome. The presently described protocol took 122–127 days for the regeneration of putatively transformed pigeon pea.

Finally, in vitro insect feeding bioassays were carried out with larvae from the major lepidopteran insect pest *H. armigera*, which showed that the Syn Cry1Ab toxin present in the green leaves of transgenic plants offered significant protection against insect-inflicted damage when compared with the control untransformed plants. In this experiment, four transformants revealed that all the transformants could resist damage caused by insects to some degree. This small variation within the four transgenic plants in the protection against insect damage can be attributed to the differences in the expression levels of the *syn cry1Ab* gene. The transgenic lines SA3, SB6, SC3, and SD1 showed a drastic size reduction of approximately 79.1–85.5%, leading to the death of 70–90% of the insects within 6 days of incubation. Similarly, Ghosh et al^9^ observed up to 100% mortality of second instar larvae at the end of insect feeding bioassays (6 days after feeding) of T_1_ transgenic plants carrying the *cry1Ac* and *cry2Aa* insecticidal genes separately^9^. The deviation in the percentage of larval mortality from our findings might be due to the amount of leaves used for the experiment, the *Bt* gene used, and the time of data collection, per the agreement with previous observations^28,29^. The present insect bioassay experiment showed a significant reduction in larval weight followed by mortality compared to larvae fed on untransformed control plants, revealing the expression of an adequate amount of toxin protein.

Antibiotic or herbicide-resistant marker genes play a crucial role as plant selection markers in the process of transgenic plant development. However, retaining the selection marker within the genome of transgenic plants poses a significant obstacle to the development of consumer-friendly GM crops. The reason for this obstacle is that the transgene could horizontally transfer from plants to bacteria or from plant products consumed as foods to intestinal microbes, with the potential for emergence of antibiotic resistance in those organisms^30^. Therefore, the routine use of plant transgenic technology may be efficient in broad-spectrum pest mortality, but the biosafety of such transgenic plants remains a critical environmental concern. Therefore, there is a need to strategically assemble these genes to develop marker-free GM pigeon pea with broader acceptance by the consumer. Extensive research has been done to develop insect-resistant pigeon pea, but until now, no marker-free plant has been generated. Thus, in the current experiment, an attempt was undertaken to develop a marker-free pigeon pea plant using Cre/*lox* technology with insecticidal activity^31,32,33,34^. To perform this, we also developed a *cre* line with a pHC construct and crossed this line with the *cry1Ab* line. As an outcome of this experiment, we obtained marker-free pigeon pea plants with an average recombination frequency of 34.52%; the highest recombination frequency was achieved in the SB6CC hybrid (35.71%). The variation in the recombination frequency between the two lines could be due to the positional effect of the transgene (*cre*) influencing the expression level, resulting in different recombinase activity^35,36^.

According to Kalve and Tadege^24^, the low success rate in the production of hybrid plants (chickpea) is due to the lack of detailed information on the flowering stages chosen for crossing. Before starting the crossing events between transgenic plants carrying *cry1Ab* and *cre* genes, we performed microscopic analysis of flowers in different stages to understand the exact timing/stage of pollination. In our present investigation, we found that stage 2 was best for emasculation (became female) and stage 3 was appropriate for collecting pollen for crossing. Furthermore, we have also observed that stage 3 is the scientifically accurate stage for crossing^24^. The reason behind this selection is that the stigma in stage 3 showed the highest pollen acceptability, as at this stage, stigma became the most sexually active for pollination, resulting in hybridization^37^. Stage 2 is the right time for emasculation because the anther starts to burst, so there is a chance of gating the stigma without pollination. This type of microscopic analysis of the developing reproductive organ combined with precise crossing was performed for the first time in pigeon pea, which helped to increase the crossing efficiency^24^.

To develop the marker-free plant, two independent T_1_ plants (SB6 and SD1) harbouring pLBRCAb (as the female parent) were crossed with a T_1_ progeny plant (CC) harbouring pHC (as the male parent). The generated hybrid plants were designated T_I_F_1_ and did not show any phenotypic aberration due to the introgression of the *cre* transgene, indicating the feasibility of efficient recombination. Excision of the selectable marker gene from the *syn cry1Ab* expression cassette could not generate complete marker-free plants, as the *cre* and *hpt*II genes remained in T_1_F_1_ hybrid plants. Further elimination of redundant transgenes was achieved via genetic segregation in the following generation (T_1_F_2_) through self-pollination. The successful complete marker gene elimination event (*syn bar* and *cre-hpt*II linked genes) was confirmed by PCR analysis. Furthermore, an insect feeding assay on T_1_F_2_ transgenic plants showed the toxicity to insects, which was responsible for inhibiting growth and led to the death of larvae. Thus, the present study was successful in generating insect-resistant genetically modified pigeon pea plants without any antibiotic resistance marker or unnecessarily present transgenes, such as *cre recombinase*.

In conclusion, the present study could be considered a breakthrough in legume/pulse research concerning the establishment of marker-free insect-resistant pigeon pea plants free of other redundant genes, which makes it one step closer to commercialization.

## Materials and methods

Experiments performed in the present study were conducted at the Advanced Laboratory for Plant Genetic Engineering, IIT-Kharagpur, West Bengal, India. The materials and methods of this work are elaborately described in the following section.

### Plant material

For the present experiment, seeds of the *C. cajan* cultivar Asha (ICPL-87119) were obtained from Indian Institute of Pulses Research, Kanpur, India. The study complies with the National guidelines for using biological materials. Permission to use the Pigeon pea seeds in the study was obtained from ICAR, Govt. of India.

### Bacterial strain

*Agrobacterium*-mediated plant transformations were carried out by the supervirulent EHA 105 strain carrying pCAMBIA1300.

### Insect source

Eggs of *H. armigera* were purchased from the NBAIR (National Bureau of Agricultural Insect Resources), Bangalore, India, to perform insect bioassay experiments with second instar larvae under laboratory conditions.

### Construction of the plant transformation vectors pLBRCAb and pHC

For the preparation of the transformation cassette, the synthetically prepared herbicide-resistant *syn bar* gene was PCR-amplified from pBAR^17^, and primers (Supplementary Table S1) were designed to have a *loxP* sequence in direct orientation. Then, the amplified *syn bar* gene flanked by the *loxP* sequence under the control of the CaMV35S prompter and terminator was cloned (between *Xho*I sites) into pCAMBIA1300 by replacement of the *hpt*II gene to obtain pLB. A lepidopteran insect pest-specific toxin gene (synthetic *cry1Ab* prepared in our laboratory^38^) was cloned under the rubisco small subunit promoter (*rbcS*) and *nos* terminator in the pCAMBIA 1300 binary vector (between *Kpn*I and *Xba*I) and named pLBRCAb (Supplementary Fig. S1a). The chimaeric gene construct was digested with the respective enzyme and analysed by agarose gel electrophoresis to confirm the presence and proper arrangement of the molecular components (Supplementary Fig. S1b). The construct of the *cre* gene cassette was prepared by cloning the *cre* gene in pCAMBIA1300 at the *Sac*I and *Kpn*I sites, and the CDS of the *cre* gene was PCR-amplified from the pX6-GFP plant DNA expression vector (Gene Bank AF 330636.1) by CRFP1/CRRP1 primers (FP: 5′ggccggtaccatgtccaatttactg3′/RP: 5′gaccgagctcctaatcgccatcttc3′) (Supplementary Table S1). The 2xCaMv35s promoter was cloned at the 5´ end of *Hind*III, the *Kpn*I site and *nos* gene were incorporated at the 3′ end of the *cre* gene at the *Sac*I and *Eco*RI sites, and the construct was named pHC (Supplementary Fig. S2a). The prepared chimaeric gene construct was digested with the respective enzyme and analysed by agarose gel electrophoresis to confirm the presence and proper arrangement of the molecular components (Supplementary Fig. S2b).

### Generation of pLBRCAb and pHC plant lines (T_0_ and T_1_)

For the generation of two separate pigeon pea lines, pLBRCAb and pHC, a well-established *Agrobacterium*-mediated transformation and regeneration protocol was used as described by Sarkar et al^17^. Well-grown regenerated transformed plants were selected in selection medium with appropriate doses of drugs (bialaphos at 4 mg/L for pLBRCAb and hygromycin at 50 mg/L for the pHC plant line). After that, surviving plants with elongated roots were exposed to hardening, transferred to the greenhouse, and allowed to grow to maturity. The individual transgenic line (T_0_) harbouring the expression cassettes pLBRCAb and pHC was allowed to self-fertilize under controlled conditions (to prevent cross-pollination) to obtain T_1_ seeds. T_1_ seeds obtained from self-fertilized individual T_0_ plants of two separate lines were screened for antibiotic sensitivity on the respective drug-supplemented media. Two-week-old seedlings were scored for resistance or sensitivity. The segregation pattern of the transgene in T_1_ progeny plants was calculated and validated by comparing the data with the expected ratio by χ^2^-test.

### Dot blot analysis

For the initial sorting of T_1_ progeny plants, dot blot analysis was performed. Total genomic DNA from young fresh leaves of transgenic plants was extracted following the CTAB method^39^ and transferred to the Hybond-N^+^ nylon membrane (GE Healthcare, US) using a dot blot apparatus (Bio-Rad Laboratories, USA). Hybridization, followed by the development of blots, was performed using a *cry1Ab* gene-specific probe according to the steps described by Sarkar et al^17^.

### Southern blot analysis

Thirty-day-old dot blot-positive transgenic plants along with one untransformed control were selected to isolate total genomic DNA from young and fresh leaves and subjected to Southern blot hybridization, according to Sambrook et al^40^. Approximately 10 μg of genomic DNA from all plants was digested with a restriction enzyme (*Hind*III) in the respective buffer. Completely digested DNA samples were electrophoresed on a 0.8% (w/v) agarose gel and subsequently transferred to Hybond-N^+^ nylon membranes using a vacuum blot transfer apparatus (Bio-Rad Laboratories*,* model 785, USA) with 20X SSC for 2 h at 4 mmHg pressure. After complete transfer of DNA, the blot was washed with 6X SSC, air-dried, and UV cross-linked in a Crosslinker, according to the manufacturer's instructions (Amersham, UK). Moreover, the PCR-amplified product of the target gene (800 bp for *syn cry1Ab* and *cre*) was radiolabelled to prepare the probe. The purified DNA was radiolabelled with α-[32P] dCTP (3500 Ci/mmol) by random priming using a DNA labelling system, according to the manufacturer's protocol (Thermo Scientific, US). After labelling, the probe was purified by passing through a Sephadex G-50 column to remove free radionucleotides. Prehybridization and hybridization of Southern blots were performed under stringent conditions of 65 °C for 2 h and 18 h, respectively, in a roller tube inside the hybridization incubator/shaker (Amersham Biosciences, UK) with Church buffer. The blots were washed with under stringent conditions as follows: 20 min in SSC (2×) and SDS (0.1%) at 50 °C; 20 min in SSC (1×) and SDS (0.1%) at 55 °C; 15 min in SSC (0.5×) and SDS (0.1%) at 60 °C; and another 15 min in SSC (0.1×) and SDS (0.1%) at 65 °C. Autoradiographic exposure was performed at room temperature by exposing the membrane to Super-Resolution Screen (Perkin Elmer) inside a Hypercassette (Amersham Life Science, UK) for 5–30 min depending on the radioactive count. The screen was scanned in the Storage Phosphor System (Cyclone Plus, Perkin Elmer) at a 300 dpi resolution using a medium carousel type to generate the autoradiographic image.

### Western blot analysis

Approximately 50 mg leaf tissues of the transgenic lines (30-day-old plants) and untransformed control plants were collected for protein extraction using extraction buffer [50 mM Tris–Cl (pH 8.0), 10 mM PMSF, 2 mM EDTA, 10 μM leupeptin, 0.02% Triton-X, and 1 μM pepstatin], and the protein concentration was estimated according to the Bradford method (Bradford 1976). Approximately 30 μg of protein was loaded for electrophoresis onto 12% SDS-PAGE gels and blotted to Hybond-N^+^ nylon membranes by a wet transfer procedure. The polyclonal antibody was raised in rabbits against bacterially expressed Cry1Ab^38^, and rabbit polyclonal anti-Cre recombinase antibody (Product no. ab41104, Abcam, UK) was used as a 1° antibody at a 1:1000 dilution. Anti-isotype IgG-horseradish peroxidase (HRP) conjugate (Sigma Aldrich, USA) was used as a 2° antibody at a 1:2000 dilution. This whole procedure was carried out using the Western Chromogenic Kit (Roche Applied Science, Germany) according to the manufacturer's protocol.

### Insect feeding bioassay under laboratory conditions

The insecticidal activity of Southern-positive T_1_ transgenic plants expressing Cy1Ab protein was analysed through a leaf-feeding bioassay using second instar larvae of *H. armigera*. Fully open leaves of thirty-day-old pigeon pea plants were incubated with larvae of *H. armigera*. Each experiment was repeated two times with five replicas, and each replicate was carried out with two larvae. The body weight reduction of larvae was recorded at 24 h intervals, and the percentage of mortality was scored after 6 days of incubation.

### The crossing of T_1_ parental transgenic pigeon pea lines for Cre/***lox***-mediated marker elimination

The T_1_ progeny plants harbouring the *cry1Ab* gene cassette were considered the female parent. Emasculation was performed during the early morning between 5 and 7 AM. The emasculation of the anther in the appropriate stage is very important; thus, all the flowering stages were critically examined, and an unopened flower at the early stage of anthesis (stage 2) was selected. The anthers present inside the flowers were removed by a needle without damaging the gynoecium. Then, emasculated flowers were covered with fine-mesh nylon cloth bags to prevent contamination from foreign pollen. Thus, the female parent was generated. On the other hand, the T_1_ progeny plant, harbouring the *cre* gene cassette with a moderate level of Cre recombinase expression, was considered the male parent. At approximately 1–3 PM, the flowers (stage 3) of the cre plant line were taken for crossing. The fine-mesh nylon cloth bags, which covered the emasculated female parent, were removed to expose the sigma, and then the staminal column of the pollen bud was used to brush pollen on the stigma of the female to disperse the pollen. Then, fine-mesh nylon cloth bags were again closed with proper tagging. The T_1_F_1_ hybrid progenies were derived from crossing between T_1_ pHC and pLBRCAb lines; consecutive generations were designated T_1_F_2_. The harvested seeds from each independent crossing experiment were collected and stored with proper labelling. Thereafter, per the requirements of our experiment, surface-sterilized seeds were allowed to germinate on MS medium in vitro followed by transfer to the greenhouse for further growth.

### Polymerase chain reaction (PCR) analysis

Genomic DNA was extracted using the CTAB method from immature leaves of transgenic pigeon pea plants to conduct PCR. The PCR amplifications for various lines were performed in separate reaction tubes using gene-specific primers for each gene. The PCR conditions and thermal profiles were performed according to the steps reported by Sarkar et al^17^. Gene-specific forward and reverse primers (Supplementary Table S1) were designed to amplify specific transgenes. The amplification products of the respective genes were verified in an EtBr-stained agarose gel (1%) and observed on a transilluminator.

## Supplementary Information

Supplementary Information.
