# Supplementary material for: Development of marker-free transgenic pigeon pea (Cajanus cajan) expressing a pod borer insecticidal protein
Source: Sci Rep. 2021 May 18;11:10543. doi: 10.1038/s41598-021-90050-8 (PMC8131364; doi:10.1038/s41598-021-90050-8)
Supplement: Supplementary file 1 — Supplementary Information. [file 41598_2021_90050_MOESM1_ESM.pdf]

# Development of marker-free transgenic pigeon pea (*Cajanus cajan*) expressing pod borer resistant insecticidal protein

**Snehasish Sarkar[1], Souri Roy[1], Sudip K. Ghosh[1, 2].**

[1]Advanced Laboratory for Plant Genetic Engineering (ALPGE), IIT-Kharagpur, Kharagpur, India.
[2]Department of Biotechnology, IIT Kharagpur, Kharagpur, India.

***Corresponding Author:** Prof. Sudip K. Ghosh, Department of Biotechnology, IIT-Kharagpur, Kharagpur-721302, India.
**E-mail:** sudip@bt.iitkgp.ac.in
Phone (Office): +91-3222-283768

a

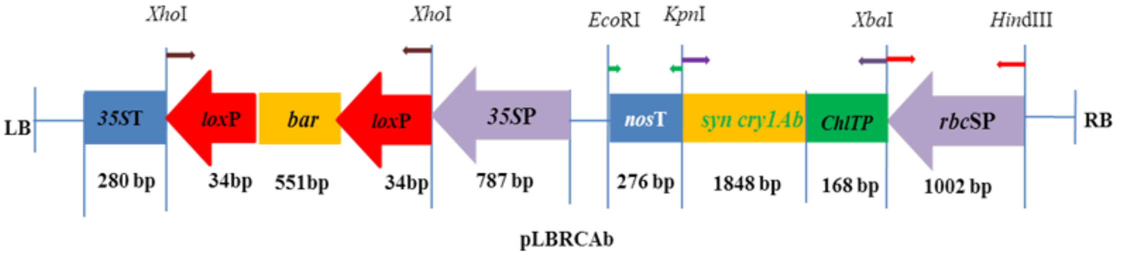


b

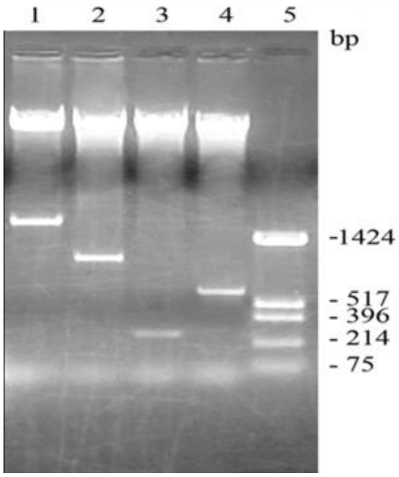


**Figure S1. Constraction pLBRCAb vector for pigeon pea transformaton: a** Schematic map of T-DNA region of binary vectore pLBRCAb carring pCAMBIA1300 *chlTP/ rbcSP/ nosT/ loxP-bar-loxP*. Arrow indicates specific primer pairs. **b** Agarose gel (1%) showing the digestion profile of molecular cassette, Lane 1: *cry1Ab* gene *(Xba*I/ *Kpn*I), lane 2: *rubisco* gene promoter *(Hin*dIII/ *Xba*I), lane 3: *nos* terminator *(Kpn*I/ *Eco*RI), lane 4: *bar* gene *(Xho*I), lane 5: *Hin*fI digested pUC18 as molecular weight marker.

a

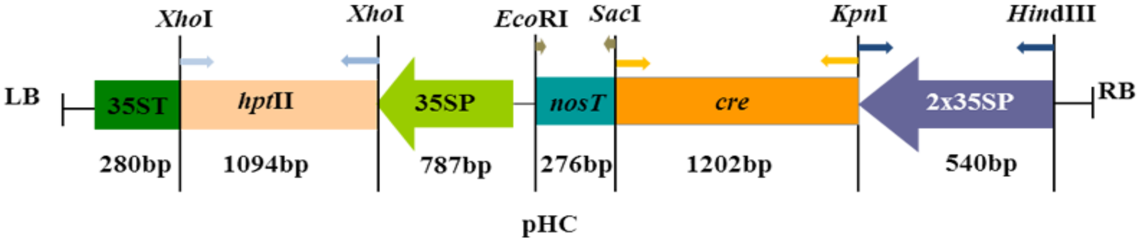


b

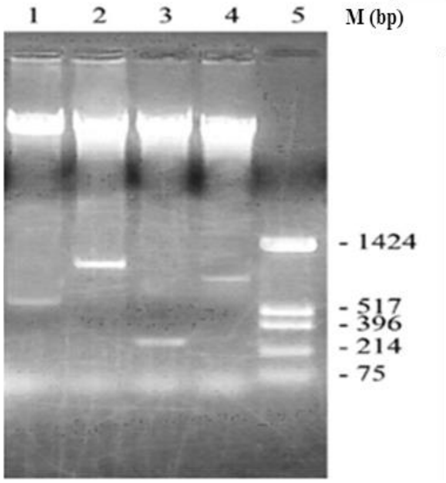


**Figure S2. Construction of the pHC vector harboring *cre* recombinase gene under constitutive promoter element for pigeon pea transformation**. **a** schematic map of T-DNA region of binary vector pHC carrying pCAMBIA1300 2x35S/*cre*/35S/*hpt*II. Arrow indicates specific primers pair. **b** Agarose gel (1%) showing digestion profile of the molecular cassette, Lane 1: 2x35S promoter, lane 2: *cre* recombinase gene, lane 3: Nos terminator; lane 4: *hpt*II gene and lane 5: *Hin*fI digested pUC18 as a molecular weight marker.

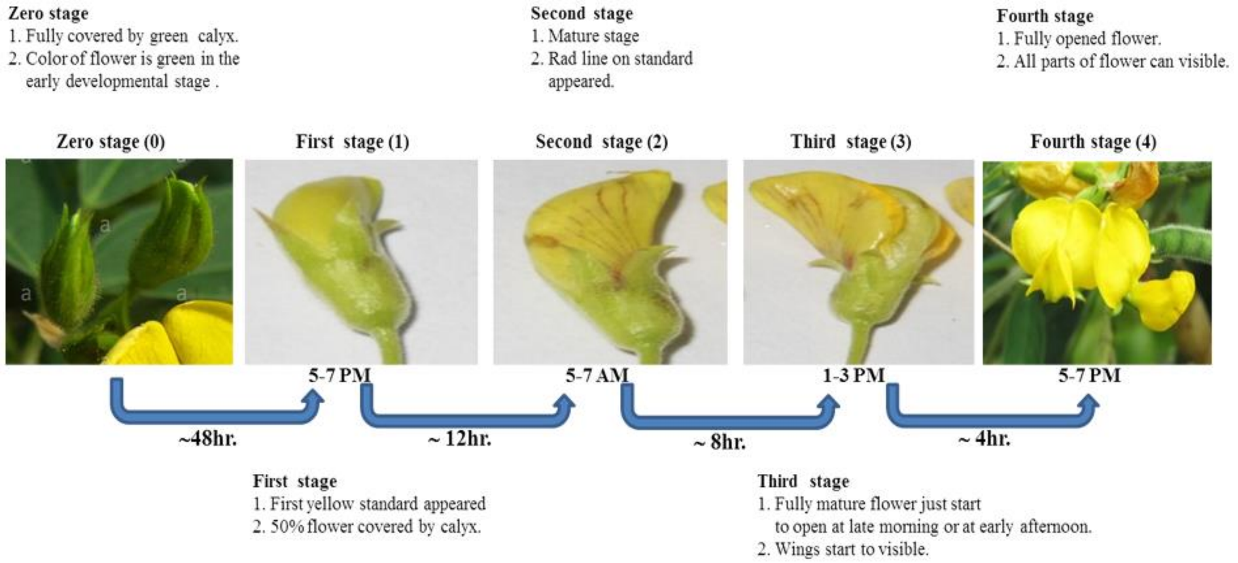


**Figure S3. Different stage of growth of pigeon pea flower in time progress and their morphological characteristics.**

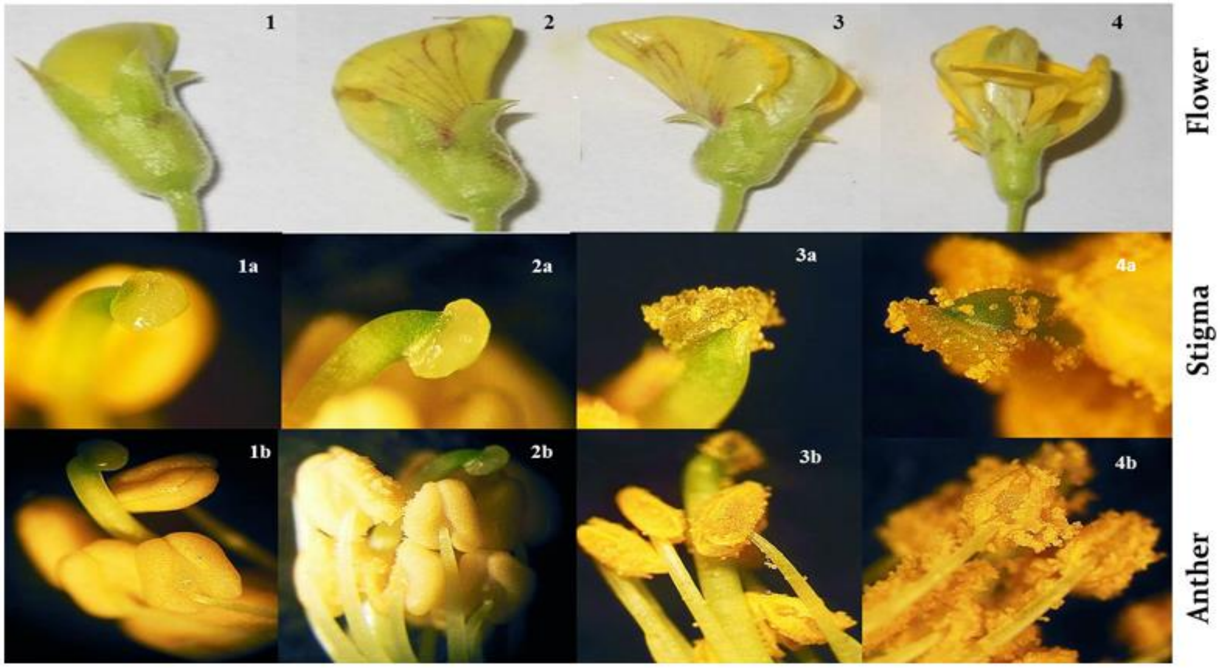


**Figure S4. Anatomical view of anther and stigma of respective pigeon pea flower in different growth stages under the light microscope**: Stage 1, 2, 3 and 4 is the flower of different growth stages, and 1a, 2a, 3a and 4a is the stigma of that respective flower. 1b, 2b, 3b and 4b represent the anther of that flowering stage. 2a, mature stigma without pollen grain attached; 2b, an anther just after the pollen release; 3a, stigma fully covered with pollen grain and 4a, stigma with few pollen grain.

**Figure S5.** Uncropped blot of Fig. 2b

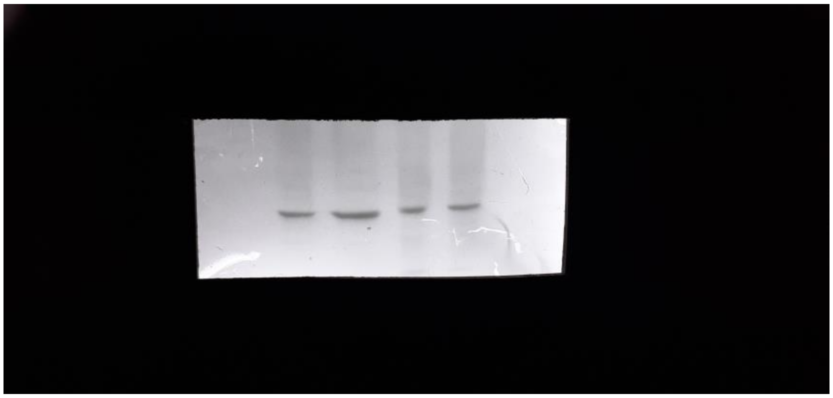

**Figure S6.** Uncropped blot of Fig. 5b

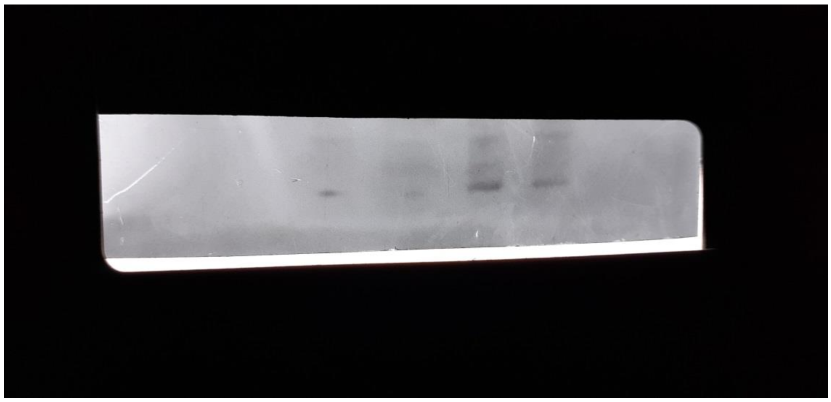

## Oligonucleotides used for PCR reactions

The detailed information of oligonucleotides, used in the study is provided in Table S1.

**Table S1:** Oligonucleotides used in the study

| Primer | 5' to 3' Nucleotide sequence | $T_m$ | Purpose |
|---|---|---|---|
| **CRFP1**<br>**CRRP1** | **FP:** ggcc**ggtacc**atgtccaatttactg<br>**RP:** gacc**gagctc**ctaatcgccatcttc | 62.0<br>63.0 | **Full gene**<br>PCR amplification of full-length *cre* gene |
| **CRFP2**<br>**CRRP2** | **FP:** cgtactgacggtgggagaat<br>**RP:** gtcatccttagcgccgtaaa | 53.8<br>51.8 | **Partial gene**<br>PCR amplification of *cre* gene |
| **BRFP**<br>**BRRP** | **FP:** ttta**ctcgag**atgagcccagaacgacg<br>**RP:** atta**ctcgag**ttatcagatctcggtgacgg | 63.0<br>61.0 | PCR amplification of *bar* gene |
| **ABFP**<br>**ABRP** | **FP:** tcccgggtgagttgtgaaacgg<br>**RP:** tcccgggtgagttgtgaaacg | 58.6<br>56.3 | PCR amplification of *cry1Ab* |
| **HPFP**<br>**HPRP** | **FP:** gtcctgcgggtaaatagctg<br>**RP:** gatgttggcgacctcgtatt | 59.92<br>59.58 | PCR amplification of *hpt*II gene |
